# Periphery kinetic perimetry: clinically feasible to complement central static perimetry

**DOI:** 10.1186/s12886-021-02056-5

**Published:** 2021-09-23

**Authors:** Xiaoxiao Ma, Li Tang, Xiaoming Chen, Liuzhi Zeng

**Affiliations:** 1 Department of Ophthalmology, West China Hospital, Sichuan University, Chengdu, Sichuan China; 2 Department of Ophthalmology, Chengdu First People’s Hospital, Chengdu, Sichuan China

**Keywords:** Glaucoma, Visual fields, Automatic kinetic perimetry

## Abstract

**Background:**

Existing evidence suggests that visual field defect in eyes with glaucoma significantly varies between individuals. The following study compared the central visual field defects with the peripheral visual field defects in patients with suspect glaucoma and primary open-angle glaucoma (POAG) and investigated whether using the central visual field test alone could result in loss of clinically valuable information.

**Methods:**

In this prospective observational study, 167 eyes from 89 patients with suspect glaucoma or POAG were first examined with static automated perimetry (SAP), followed by a peripheral visual field test on Octopus 900 perimeter (Haag-Streit, Koeniz, Switzerland). The peripheral visual field test was performed by “Auto Kinetic Perimetry” program, in which Goldmann III4e stimuli randomly moved along 16 vectors at a constant angular velocity of 5 deg/s.

**Results:**

Glaucomatous peripheral visual field defects were seen in 18% of the eyes with a normal central visual field. In addition, 86% of glaucoma patients with moderate-to-severe central visual field defects had corresponding peripheral visual field defects in the form of localized or diffuse depression of the isopters. Furthermore, a moderate correlation was found between the central and peripheral visual fields. The median test duration was 71 s for the peripheral test and 803 s for the central test (*p* < 0.001).

**Conclusions:**

Our study demonstrated the diversity of glaucomatous visual field defects, as well as the possibility of losing the clinically valuable information due to focusing on the central visual field test alone. The peripheral kinetic perimetry is clinically feasible to complement the central static perimetry for a comprehensive assessment of visual function in glaucoma patients.

## Background

Glaucoma is the second leading cause of global blindness. It is a group of eye conditions characterized by irreversible optic nerve damage and progressive visual field defect [1–3]. Visual field testing has a key role in assessing and monitoring the visual function in patients with glaucoma. For decades, the static automated perimetry (SAP) has replaced Goldmann kinetic perimetry facilitating a more reproducible and sensitive visual field loss detection, allowing earlier detection of glaucoma [4–6]. Yet, existing evidence suggests that visual field defects in the eyes of glaucoma patients significantly vary between individuals, and no single technique has been proven to be superior in all patients [7]. Studies have suggested that the results of SAP within the central 30° are insufficient to provide complete and accurate information of the peripheral visual field [8–12]. Moreover, the central visual field tests can not reveal the multiple and complicated visual field defects expanding to the peripheral area.

The kinetic perimetry is sensitive to detect peripheral visual field defects and is correlated with daily living activities [13–16]. Goldmann kinetic perimetry, which is a high-quality assessment of the peripheral visual field, can be obtained within a short test time by a well-trained and experienced perimetrist. However, manual kinetic perimetry is technician-dependent and lacks standardization [6, 17]. On the other hand, the “Auto kinetic perimetry” program using Octopus 900 perimeter provides greater standardization and shorter test time of the peripheral visual field test.

The purpose of this study was to compare the results obtained with the central static and peripheral kinetic visual field tests in patients with primary open-angle glaucoma (POAG). Furthermore, the study investigated whether clinically valuable information is lost by solely focusing on the central visual field test.

## Methods

This prospective observational study was approved by the Ethics Committee of the West China Hospital of Sichuan University, and it adhered to the principles of the Declaration of Helsinki. Written informed consent was obtained from each individual after explaining the study design and procedure in detail.

### Subjects

A total of 167 eyes of 89 consecutive patients (48 men and 41 women) were recruited and prospectively evaluated at the eye clinic of the West China Hospital of Sichuan University. All patients were diagnosed with POAG or glaucoma suspect. Diagnosis of POAG was based on the presence of glaucomatous optic nerve head (ONH), open anterior chamber angle, and visual field defects corresponding to ONH appearance, regardless of the level of intraocular pressure (IOP). Glaucoma severity was staged based on the SAP using the Hodapp-Parrish-Anderson criteria [18]: early glaucoma (mean deviation (MD) less than − 6 dB), moderate glaucoma (MD ranging from − 6 to − 12 dB), and severe glaucoma (MD greater than -12 dB). Glaucoma suspect was defined as suspicious-appearing optic discs and/or ocular hypertension (IOP > 21 mmHg) without repeatable glaucomatous visual field defect.

The inclusion criteria included: i) patients between 16 and 70 years old; ii) best-corrected visual acuity (BCVA) of 20/40 or better; iii) spherical ametropia within ±6 diopters (D); iv) cylindrical ametropia within ±2D; v) sufficient cognitive and motor ability to perform the tests. All patients had experience in the automated static visual field testing; however, none of them underwent kinetic perimetry earlier.

Exclusion criteria were: i) patients with concurrent eye diseases affecting the visual field, e.g., amblyopia, strabismus, ocular motility disorder, macular degeneration, retinal vein or artery occlusion, diabetic retinopathy, drusen of the optic nerve head, and other neuro-ophthalmological diseases; ii) relevant opacities of the central refractive media (cornea, lens, vitreous body); iii) the use of miotic drugs or any other drugs that may affect the visual field test; iii) any intraocular surgery (except uncomplicated cataract surgery, more than 3 months previous to testing); iv) medical history that may affect the visual field, i.e. smoking, a history of pituitary adenoma or other systemic disorders that could result in visual field defects

### Examinations

All participants underwent comprehensive ophthalmic examinations, including Snellen decimal BCVA IOP (TX-20 non-contact tonometer, Canon, Japan), slit-lamp anterior segment examination, magnified stereoscopic visualization of the fundus obtained with the slit lamp, and manifest refraction. Both static and kinetic visual field tests were performed with the Octopus 900 Perimeter (Haag-Streit, Koeniz, Switzerland), which included a full-size Goldmann spherical cupola covering the entire 90-degree visual field area. All tests were performed on the same day. The central visual field test was conducted first; after a 10-min rest break, the peripheral visual field test followed.

#### Central static visual field testing

SAP for this study was performed with the 32 Standard Normal programs, which consisted of 74 testing points and examined the full 30 degrees of central visual field using stimuli Goldmann III4e with stimulus duration of 100 ms. The test points were spaced 6 degrees apart. For the central static visual field testing, standard 38 mm trial lenses with full diameter aperture were used to correct the refractive errors larger than + 3.00DS, − 1.00DS, and ± 1.00 DC. Moreover, appropriate near refraction with additional adjustment for age was provided for each patient when the central visual field was measured. Fixation was steadily monitored by the automated eye-tracking program, which could automatically readjust the patients’ eye position and work with maximum fixation and blink control. During the testing session, the non-examined eye of the patient was covered by a white and translucent eye occluder.

#### Peripheral kinetic visual field testing

The peripheral kinetic visual field testing was performed with the “Auto Kinetic Perimetry” program, in which Goldmann III4e stimuli moved along 16 vectors randomly at a constant angular velocity of 5 deg/s. The background luminance was 31.4 apostilbs. These test parameters (stimulus characteristics and background illumination) were identical to those applied in Goldmann perimetry. The stimuli moved radially from the periphery towards the center. Participants were asked to press the response button as soon as the moving stimulus was perceived. The examiner supervised the fixation of each participant via a monitor. The examiner corrected the subject’s eye and head position manually if necessary. In order to avoid lens rim artifacts, the peripheral kinetic visual field testing was performed without refractive correction since previous studies showed the optical defocus had no impact on the kinetic sensitivity using III4e stimuli [19, 20]. Three repetitions were performed for each patient. All participants underwent the first peripheral kinetic visual field tests following the examiner’s instruction. Due to the learning effect [21, 22], only the results of the last two measurements were evaluated.

#### Reliability of visual field testing

We considered that the results of the central static visual field test were unreliable if the reliability factor (RF) was > 15 [23, 24]; the results of the peripheral kinetic visual field test were unreliable if the patients’ fixation was assessed as poor by the examiner or the isopter radius difference on the same vector between the two tests was > 5 degrees [17].

#### Classification of visual field results

The visual field results were independently reviewed and classified by two masked physician reviewers (Xiaoming Chen & Li Tang). The visual field results were distributed in a randomized fashion, in which the central and peripheral visual field examinations were not grouped in any order. In this way, the reviewers could not compare the examinations of one patient. The interpretations were based on predetermined criteria that placed a field into one of three categories: (1) normal, (2) localized defect, (3) diffuse defect (Tables 1 and 2) [25, 26]. The masked descriptions by each reviewer were compared for each visual field. A consensus description was obtained if the descriptions from the two reviewers had exactly the same pattern. Otherwise, the third reviewer (Liuzhi Zeng) reassessed the visual field pair in an unmasked fashion.

**Table 1:** Criteria used for interpretation of central static visual fields

Interpretation	Criteria
Normal	None of the following defects
Localized defect	≥10 dB loss at two or more contiguous points ≥5 dB loss at three or more contiguous points ≥10 dB difference across the nasal horizontal midline at two or more adjacent points
Diffuse defect	≥4 dB loss of Mean Sensitivity (MS)

**Table 2:** Criteria used for interpretation of peripheral kinetic visual fields

Interpretation	Criteria
Normal	None of the following defects
Localized defect	> 10° localized offset compared with the normal isopter
Diffuse defect	Concentric constriction of the isopter

### Statistical analysis

All the data were compiled into a Microsoft Excel spreadsheet and analyzed using the statistical software package SPSS version 26.0 (IBM SPSS Statistics, Chicago, USA). Paired t-test was used to determine whether there was a significant difference between the two measurements of the peripheral visual field test. The test duration of both examinations was measured in seconds and compared using a Wilcoxon rank-sum test. Spearman rank-order correlation was used to examine the relationship between the central and peripheral visual fields. A *P* value < 0.05 was considered to be statistically significant.

## Results

A total of 167 eyes of 89 patients (48 males and 41 females) with POAG or suspect glaucoma were recruited in the study. The general characteristics of the participants are provided in Table 3. The median test duration of the peripheral kinetic visual field tests was 71 s, which was significantly shorter compared to the central static visual field tests (*p* < 0.001). No significant differences were found in the isopter area between the two measurements of peripheral kinetic visual field test (*p* = 0.095), which suggests good test repeatability.

**Table 3:** General characteristics of 89 study subjects (167 eyes)

Age, Mean (SD), y	38.73 (12.57)
Age range, y	16–68
Sex, Male, No.(%)	48 (53.93)
IOP, Mean (SD^*^), mmHg	19.04 (0.36)
BCVA, Median(IQR^†^)	0.96 (0.20)
Test duration, Median(IQR), s	
Central static visual field test	803(185)
Peripheral kinetic visual field test	71(17.5)
MD, Median(IQR), dB	1.60(2.90)
MS, Median(IQR), dB	25.50(3.10)
Diffuse Defect, Median(IQR), dB	1.10(1.90)
Mean Isoper Area, Mean(SD), deg^2^	12,331.65(1549.81)

^*^SD Standard deviation

^†^*IQR* interquartile range

### Comparison of central static and peripheral visual fields

**Fig. 1:**
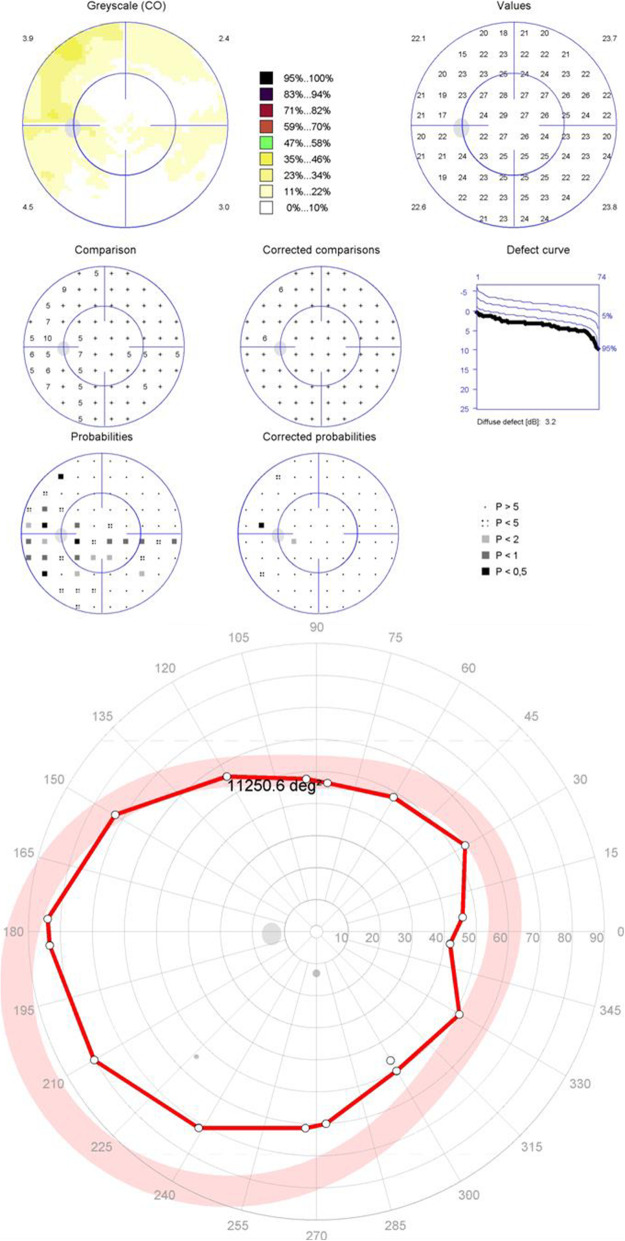
Patient 1 with normal central visual field and a depressed isopter

The results of the peripheral and central visual field tests are shown in Table 4. Among the 99 eyes with normal central visual fields, peripheral visual field defects were seen in 18 eyes (18%)(Fig. 1), peripheral tests revealed that three of the 18 cases presented concentric constriction of the isopter, while the other 15 cases had localized defects.

**Table 4:** Results of central static and peripheral kinetic visual field results

Classification of central static visual field results	Classification of peripheral kinetic visual field results, No (%) of eyes				Total, No.(%) of eyes
	Normal	Localized defect	Diffuse defect	Both localized and diffuse defect	
**Normal**	**81(82)**	**3(3)**	**15(15)**	**0**	**99(100)**
**Mild visual field Defect**	**32(70)**	**10(22)**	**4(9)**	**0**	**46(100)**
Localized Defect	29(63)	10(22)	3(7)	0	42(91)
Diffuse Defect	1(2)	0	0	0	1(2)
Both Localized and Diffuse Defect	2(4)	0	1(2)	0	3(7)
**Moderate to Severe visual field Defect**	**3(14)**	**11(50)**	**5(23)**	**3(14)**	**22(100)**
Localized Defect	3(14)	9(41)	1(5)	0	13(59)
Diffuse Defect	0	0	1(5)	0	1(4)
Both Localized and Diffuse Defect	0	2(8)	3(14)	3(14)	8(36)

For the patients with mild central visual field defects, 32 (70%) cases showed normal isopters; three cases (7%) showed localized defects in the central visual field tests but presented diffuse defects in the peripheral visual field tests. In addition, ten cases (22%) showed localized defects in both central and peripheral tests.

**Fig. 2:**
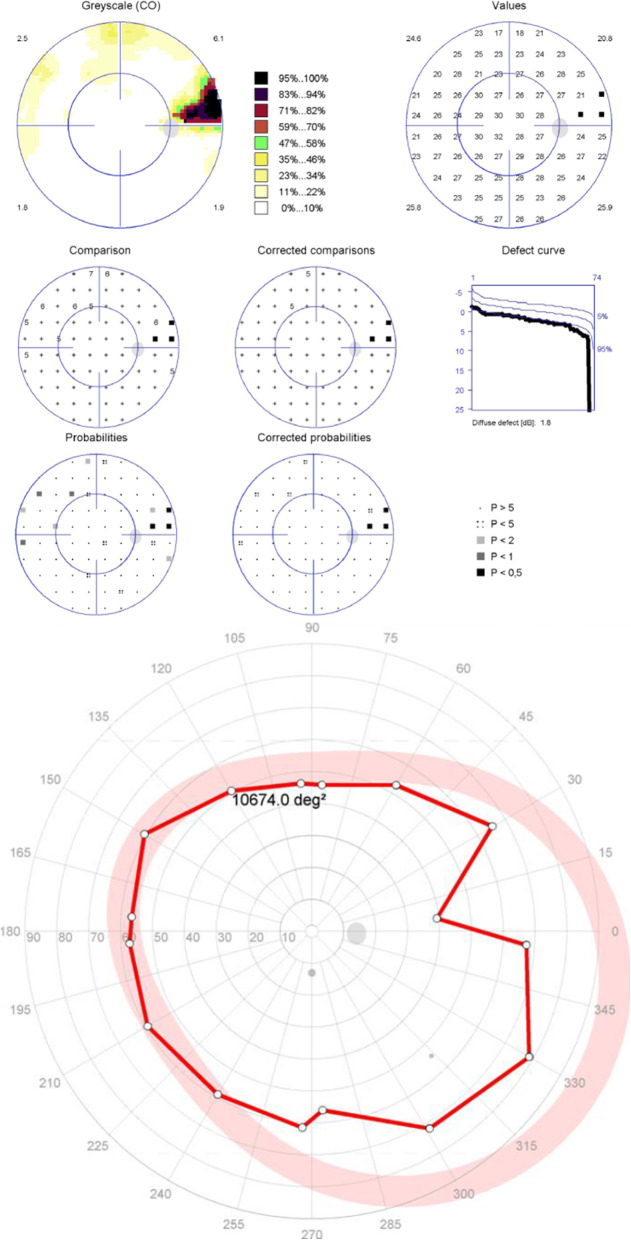
Representative example of both central static and peripheral kinetic visual field tests demonstrating localized visual field defect

**Fig. 3:**
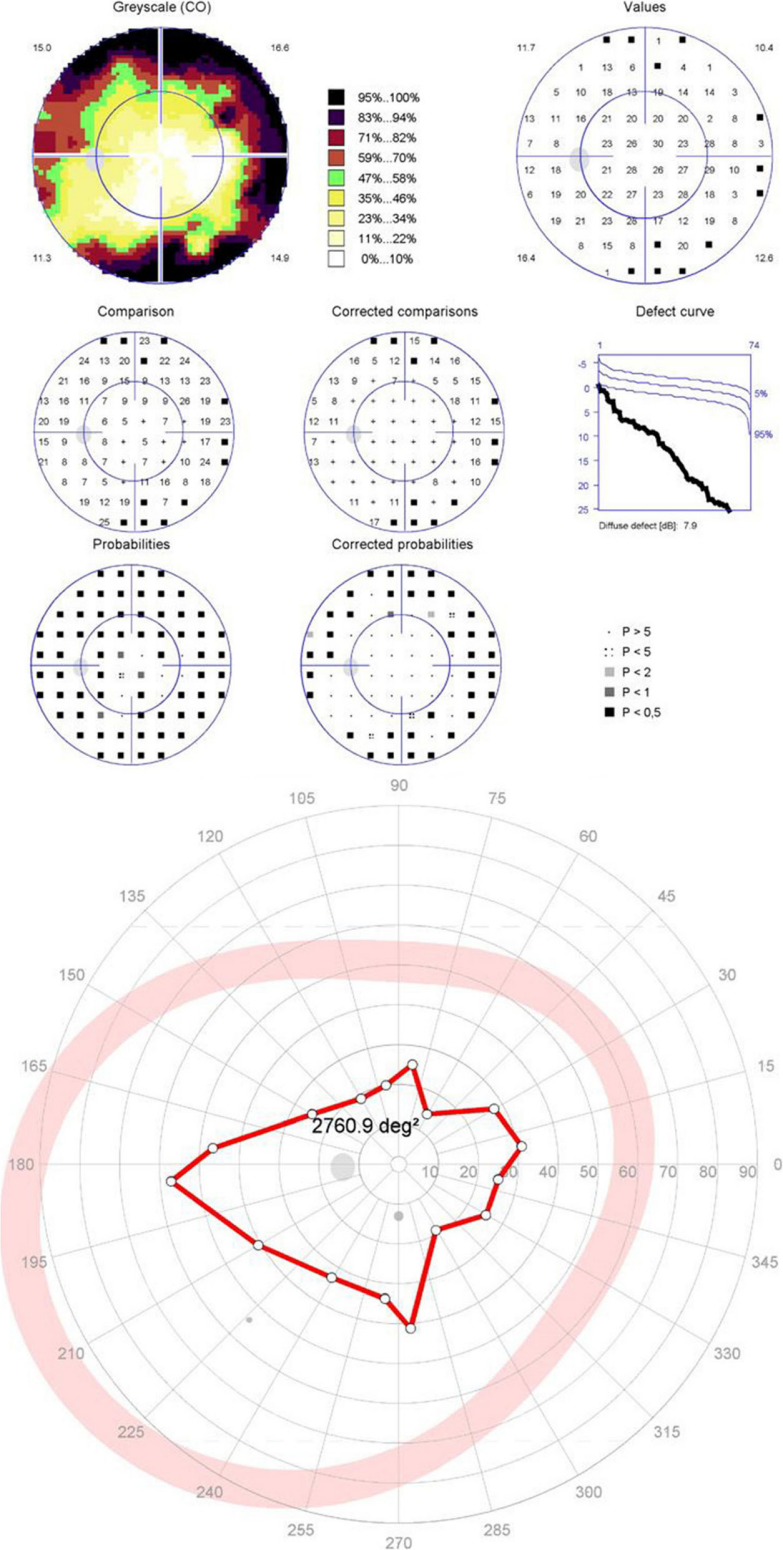
Both central static and peripheral kinetic visual field tests demonstrated diffuse visual field defects

**Fig. 4:**
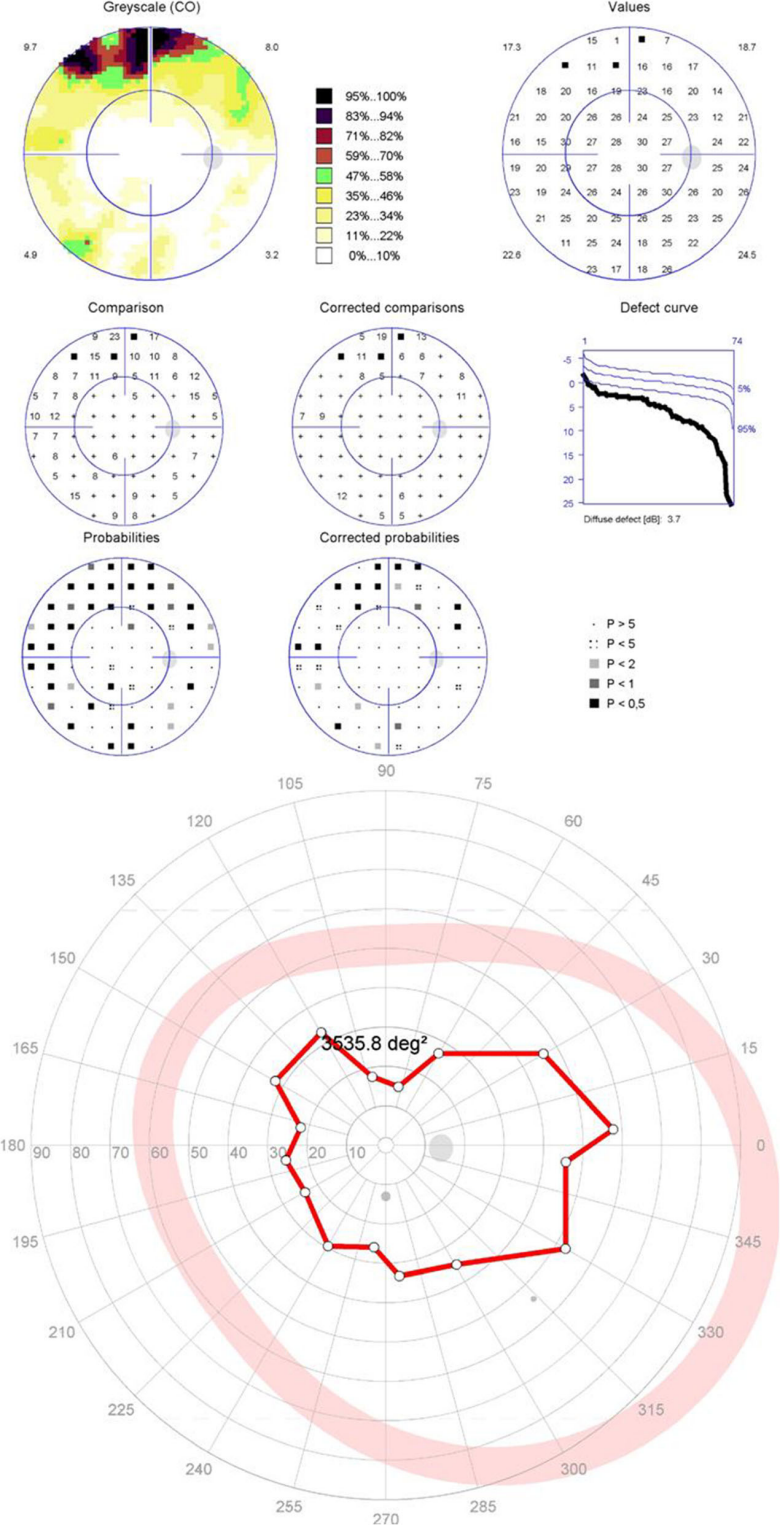
Patient 2 with a moderate localized defect in the central visual field but a diffusely constricted isopter in the peripheral visual field

**Fig. 5:**
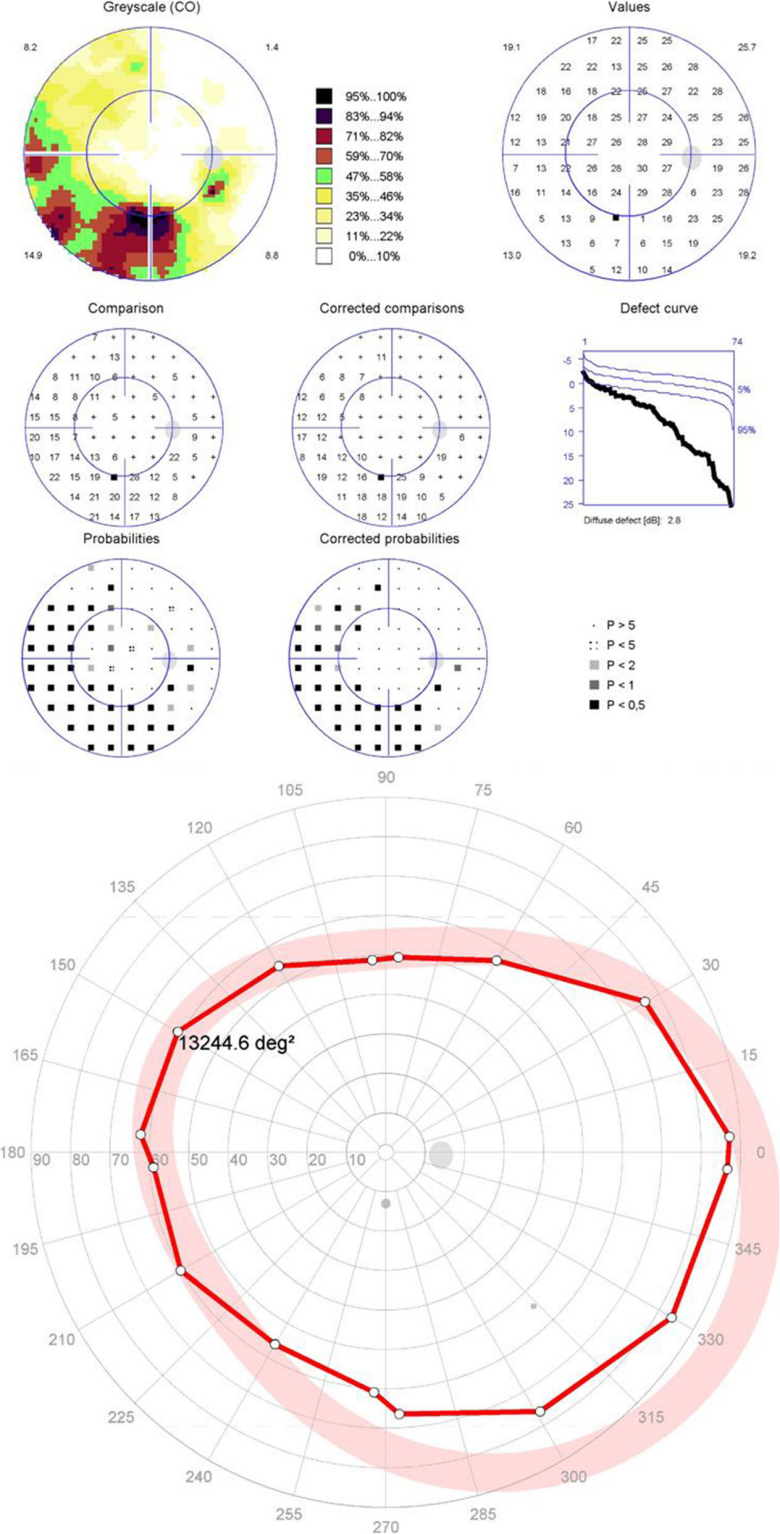
Patient 3 with a deep focal damage in the central visual field but a substantially preserved isopter in the peripheral visual field

For the patients with moderate to severe glaucomatous central visual field defects, the results of the peripheral tests supported the diagnosis made with central tests in 19 eyes (86%) (Figs. 2 and 3). The forms of the peripheral visual field defects were different from that of the central visual field defects in 9 eyes (Fig. 4). Three eyes (14%) suffered moderate to severe glaucomatous central visual field defects with nearly normal peripheral isopters (Fig. 5).

### Relationship between peripheral and central visual fields

**Fig. 6:**
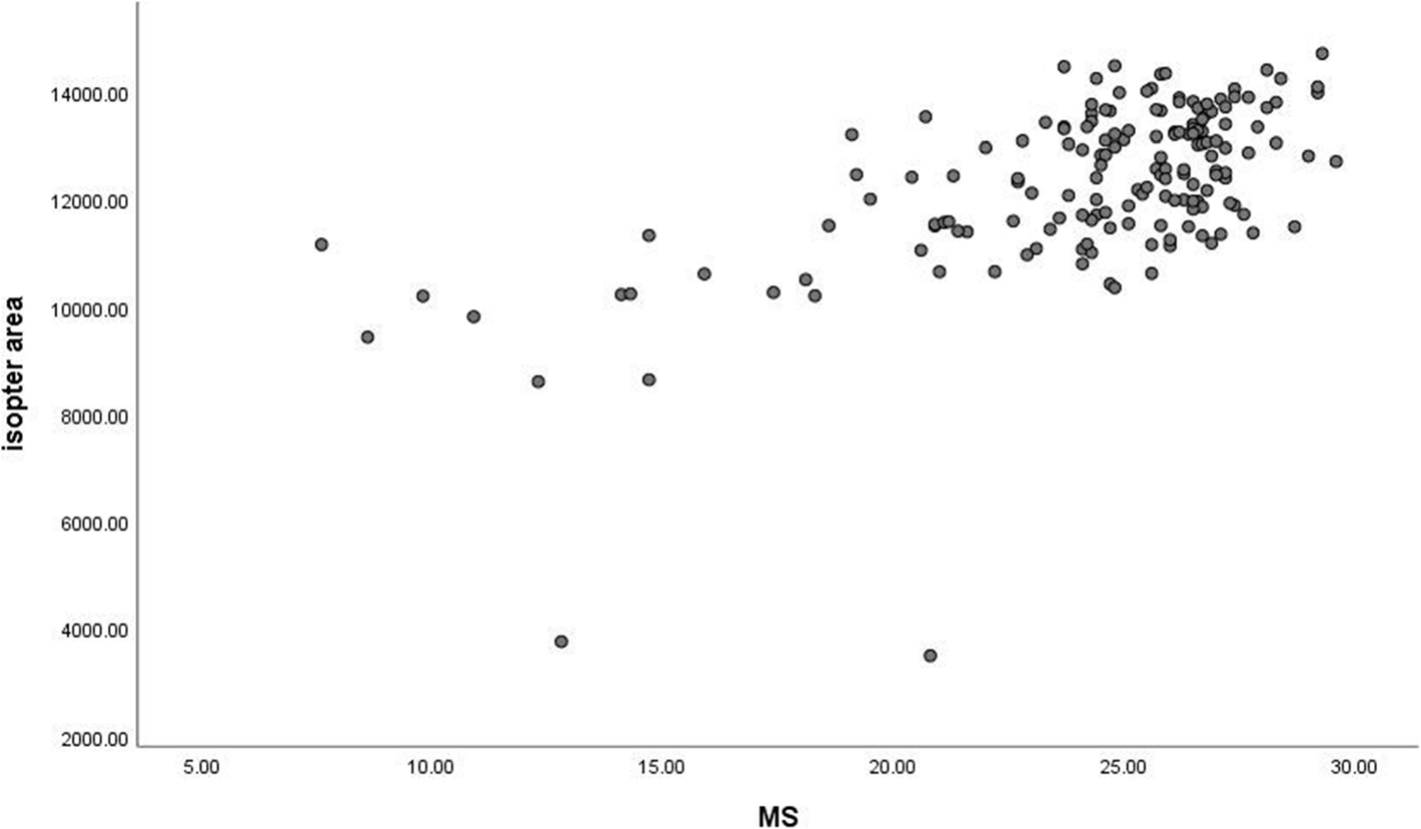
Relationship between the isopter area and MS. The Spearman rank-order correlation coefficient was 0.494 (*p*<0.001)

**Fig. 7:**
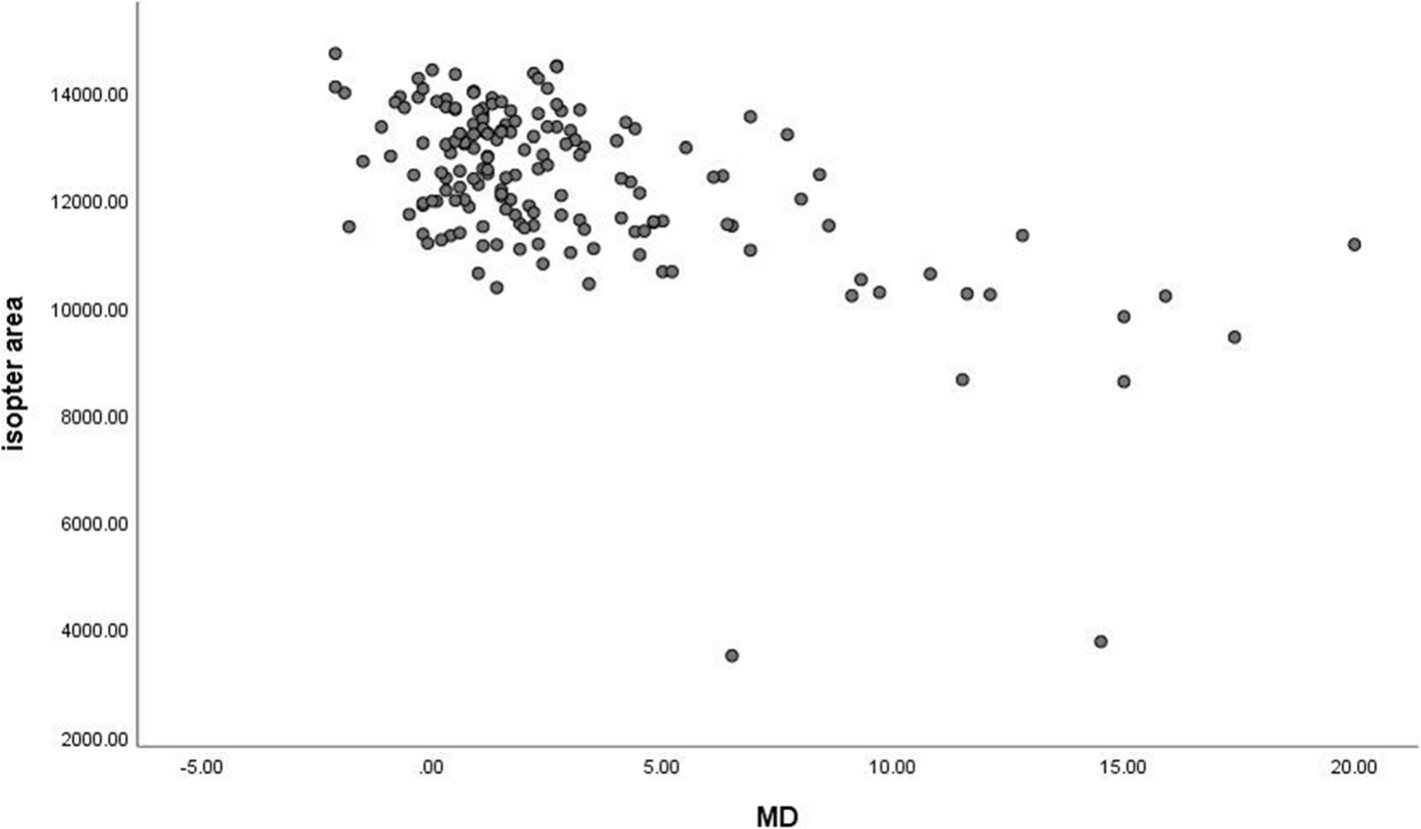
Relationship between the isopter area and MD. The Spearman rank-order correlation coefficient was − 0.446 (*p*<0.001)

**Fig. 8:**
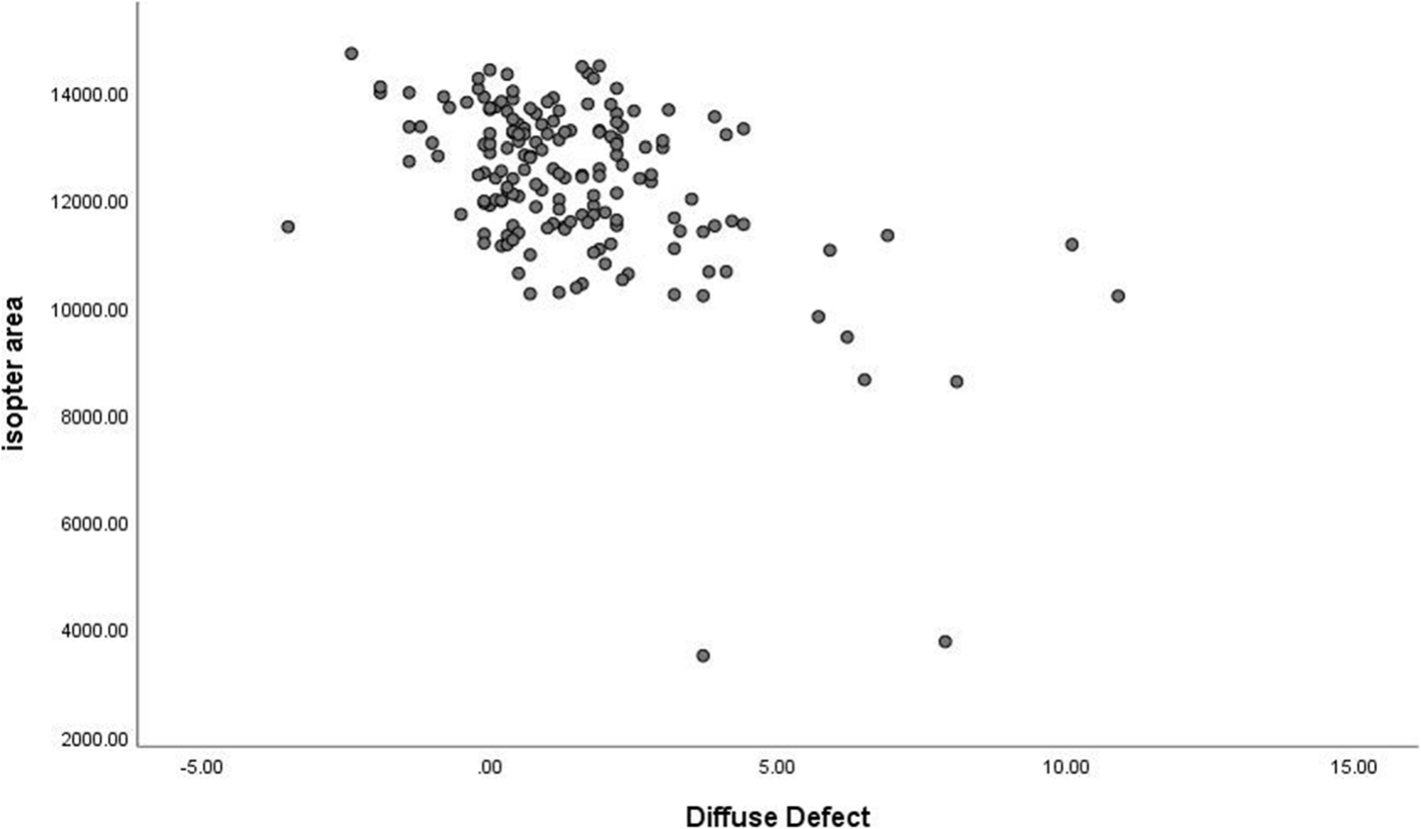
Relationship between the isopter area and diffuse defect. The Spearman rank-order correlation coefficient was − 0.375 (*p*<0.001)

MS was moderately correlated with the isopter area (rs = 0.494, *P* < 0.001) (Fig. 6). The correlation was found between the isopter area and MD (r_s_ = − 0.446, *P* < 0.001) (Fig. 7). Moreover, the correlation between the isopter area and diffuse defect was weak (r_s_ = − 0.375) (Fig. 8).

## Discussion

This study revealed the differences between the central and peripheral visual field results over a wide spectrum of glaucoma severity. We demonstrated the diversity of visual field defects caused by glaucoma. Glaucomatous visual field defects occurred throughout the field of vision in forms of localized or diffuse defects or both. Moreover, a moderate correlation was found between the isopter area and MS. One of the main advantages of our study was that relatively large areas of the peripheral visual field could be examined within a fairly short time.

In the group of glaucoma suspects and early-stage POAG patients, glaucomatous peripheral defects were observed in 18% of eyes with a normal central visual field, while 70% of the eyes had normal isopters with the glaucomatous central visual field. This suggests that the early glaucomatous visual field defects tend to occur only in the central 30° or the peripheral area, or both. The results of our study are similar to the previous observations using an automated perimeter (Fieldmaster 5000), which found glaucomatous peripheral visual field defects in 4.2% of patients with normal central fields when one isopter was used [27], and in 7% of patients when a more sensitive isopter was added [28]. In the study of automated kinetic perimetry using the Humphrey Field Analyzer, the results of peripheral visual field supported the diagnosis made with central field testing in approximately one-third of the eyes and added additional diagnostic information in another fourth of the cases [26]. Our findings support the previous work suggesting that patients with similar central visual field loss may have strikingly different peripheral visual fields [29].

In our study, 86% of the glaucoma patients with moderate to severe central visual field defects had corresponding peripheral visual field defects in the forms of localized or diffuse depression of the isopters. Our findings are consistent with the previous studies suggesting that peripheral kinetic perimetry provides additional information to SAP in assessing the remaining visual field, and thus could be used to monitor disease progression in end-stage glaucoma [10, 30]. Nowomiejska K et al showed that semi-automated kinetic perimetry (SKP) provides additional information over SAP in patients with end-stage glaucoma (this was observed in 54% cases), as defined by disc appearance (cup-to-disc ratio worse than 0.9) and SAP criteria (MD worse than 20 dB) [10]. Furthermore, kinetic perimetry is superior to static perimetry in exploring and defining the consequences of visual impairment in daily activities [13, 14, 31–33].

In the present study, we calculated the isopter area to quantify the peripheral visual field. To the best of our knowledge, this is the first study that assessed the correlation between the isopter area and SAP parameters. We found that MD, MS, and diffuse defect were correlated with the isopter area. Recently, Mönter and colleagues reported that the mean radius of the isopter (MIR) and MD were only moderately related (Spearman’s ρ, 0.51) [29]. This means that peripheral visual field tests can reflect the visual function impairment in certain cases and should not be ignored or even replaced by SAP.

No significant differences were found in the isopter area between the two measurements of peripheral kinetic visual field test (*p* = 0.095), suggesting good test repeatability. Patients preferred kinetic perimetry compared with the static test. The reason could lie in the shorter duration, brighter stimulus, and easier cooperation required by the kinetic test using the “Auto Kinetic Perimetry” program. This may alter the previous bias that a peripheral kinetic visual field test was too time-consuming (ranged from 5 mins to 15 mins), which would make its clinical application challenging [9, 26–29, 34, 35]. Furthermore, an examiner was required to monitor fixation and retest vectors if the result was considered unreliable. Thus, minimal perimetry skill was required on the part of the examiner during the test. The software calculated the area encompassed by the isopter, which can be used to monitor the progression of the disease. Finally, the use of the computerized “Auto Kinetic Perimetry” program makes the perimetry kinetic test standardized, examiner-independent, and reproducible.

This study has one limitation. We did not correct the isopters for reaction time (RT) because we did not have a permit to access the program on the Octopus 900 perimeter. The RT increases with eccentricity and age, and decreases with the growth of stimulus luminances [17]. The measurement of the RT is of special interest for subjects with severe retinal or neurological diseases and old participants who are slow to respond to the stimulus [36, 37]. However, the RT had a slight influence on our test since the patients enrolled in our study were under 70 years old and had sufficient cognitive ability a to undertake visual field tests.

## Conclusion

Our study results demonstrated the diversity in glaucomatous visual field defects. We also proved that focusing on the central visual field test alone can lead to a loss of clinically valuable information. By integrating the peripheral kinetic visual field test with the central one, it would be possible to improve the detection of early glaucomatous visual field loss in the peripheral and the evaluation of severe visual field defects. Peripheral kinetic perimetry can complement the central static perimetry and provide a comprehensive assessment of glaucoma patients’ visual function.

## Data Availability

The datasets used and/or analyzed during the current study are available from the corresponding author on reasonable request.
